# Quick and wide-range taxonomical repertoire establishment of the cystic fibrosis lung microbiota by tandem mass spectrometry on sputum samples

**DOI:** 10.3389/fmicb.2022.975883

**Published:** 2022-10-13

**Authors:** Pauline Hardouin, Olivier Pible, Hélène Marchandin, Karen Culotta, Jean Armengaud, Raphaël Chiron, Lucia Grenga

**Affiliations:** ^1^Département Médicaments et Technologies pour la Santé (DMTS), Université Paris-Saclay, CEA, INRAE, SPI, Bagnols-sur-Cèze, France; ^2^Université de Montpellier, Laboratoire Innovations Technologiques pour la Détection et le Diagnostic (Li2D), Bagnols-sur-Cèze, France; ^3^HydroSciences Montpellier, CNRS, IRD, Service de Microbiologie et Hygiène Hospitalière, Université de Montpellier, CHU de Nîmes, Nîmes, France; ^4^HydroSciences Montpellier, CNRS, IRD, Centre de Ressources et de Compétences de la Mucoviscidose, Université de Montpellier, CHU de Montpellier, Montpellier, France

**Keywords:** cystic fibrosis, proteotyping, microbiota, sputum, sampling method, metaproteomics, mass spectrometry

## Abstract

Microorganisms proteotyping by tandem mass spectrometry has been recently shown as a powerful methodology to identify the wide-range taxonomy and biomass of microbiota. Sputum is the recommended specimen for routine microbiological monitoring of Cystic Fibrosis (*CF*) patients but has been rarely submitted to tandem mass spectrometry-based proteotyping. In this study, we compared the microbial components of spontaneous and induced sputum samples from three cystic fibrosis patients. Although the presence of microbial proteins is much lower than host proteins, we report that the microbiota’s components present in the samples can be identified, as well as host biomarkers and functional insights into the microbiota. No significant difference was found in microorganism abundance between paired spontaneous and induced sputum samples. Microbial proteins linked to resistance, iron uptake, and biofilm-forming ability were observed in sputa independently of the sampling method. This unbiased and enlarged view of the *CF* microbiome could be highly complementary to culture and relevant for the clinical management of *CF* patients by improving knowledge about the host-pathogen dynamics and *CF* pathophysiology.

## Introduction

Cystic fibrosis (*CF*) is a recessive genetic disease in which mutations of the *CF* transmembrane conductance regulator (CFTR) gene result in a multi-systemic disorder. Despite a characteristic impairment of different organs, the primary cause of morbidity and early mortality in this disease is attributable to progressive airway and lung parenchymal damage through a cycle of infection, inflammation, and tissue damage (Cuthbertson et al., 2020).

Increased knowledge of airway infections in *CF* and their trajectories represents a keystone for a better understanding of the underlying *CF* pathophysiology. Their deep characterization could open new perspectives to improve their clinical management. Our understanding of the polymicrobial characteristics of *CF* airway infections and, more generally, of the *CF* respiratory microbiome is driven by pioneering studies applying next-generation sequencing (NGS)-based strategies (van der Gast et al., 2011; Quinn et al., 2014; Coburn et al., 2015; Feigelman et al., 2017). These works established the connections between significant changes in the airway microbiome composition and increased age, antibiotic use, decreased lung function, environment, and *CF* disease progression (Héry-Arnaud et al., 2019; Cuthbertson et al., 2020). However, host-pathogen interactions, microbiota dynamics, and their impact on the disease are only partially unveiled.

Besides the powerful metagenomics technology, deep shotgun proteomics represents a promising toolbox to get quicker unbiased information on protein samples, whatever their complexity. Metaproteomics, combining high-resolution tandem mass spectrometry with liquid reverse phase chromatography, has different facets: this methodology can precisely identify taxa present, estimate the respective biomasses of the components of the microbiota (Pible et al., 2020), describe how the microorganisms function by means of the detected proteins (Van Den Bossche et al., 2021a) and deliver unique functional characterization of the host molecular response (Van Den Bossche et al., 2021a; Grenga et al., 2022). Its potential to assess the lung microbiota using as starting material sputum, bronchoalveolar lavage (BAL), or epithelial brush has been recently evoked (Grenga et al., 2019; Wang et al., 2020; Hardouin et al., 2021).

Traditionally, BAL has been used to define airway microbiology and inflammation in young *CF* children. This sampling method presents an invasive character, the requirement of anesthesia, and is time- and resources- consuming. Compared to this gold standard for diagnosing lower airway microbiota, induced sputum samples showed a good bacteriologic correlation with BAL (Blau et al., 2014; Dubourg et al., 2015). Because it is a non-invasive method of sampling, the relevance of sputum for the study of the pulmonary microbiome is demonstrated (Lim et al., 2013; Hahn et al., 2016; Hogan et al., 2016; Feigelman et al., 2017; Pattison et al., 2017); (Acosta et al., 2018; Bevivino et al., 2019). Sputum is the recommended specimen for routine microbiological monitoring of *CF* patients and can be sampled either spontaneously for expectorating patients or induced in non-expectorating patients (Hogan et al., 2016; Cuthbertson et al., 2020). Thus, sputum has the advantage of possible iterative sampling for fine-tuned monitoring (Françoise and Héry-Arnaud, 2020). *CF* sputum samples are among the most challenging samples because of their heterogeneity and viscosity and the low ratio of microbial protein load (Graf et al., 2021). The latest published protocol for proteomics comprises a significant number of steps for the preprocessing of sputum, microbial enrichment, protein extraction, and proteolysis (Graf et al., 2021). Taxonomical identification of pathogens by mass spectrometry proteotyping, i.e., Matrix Assisted Laser Desorption Ionisation - Time of Flight (MALDI-ToF)-based identification, is rapidly obtainable following isolate cultivation and, over the last decade, has become a standard diagnostic tool in the microbiological laboratory (Suarez et al., 2013). A more complete and informative description of the taxon diversity can be obtained directly from the biological sample through tandem mass spectrometry-based proteotyping, avoiding culture bias (Grenga et al., 2019). Here, we assessed this methodology on sputum samples to establish the composition of the *CF* microbiota and the associated functionalities. However, as a general rule in medical microbiology, results may depend highly on the analyzed sample type and quality. Therefore, in this proof-of-concept study, we compared the respective diagnosis value of spontaneous and induced sputum samples to determine the sample providing the more accurate results using tandem mass spectrometry.

## Materials and methods

### Sputum sampling

The sampling was conducted at the Centre de Ressources et de Compétences de la Mucoviscidose in the University Hospital of Montpellier (France) following study approval from the review board of the institution (IRB-MTP_2021_02_202000665; 2021-02-18). Spontaneous and induced sputum samples were collected from three *CF* adult patients during follow-up consultation and after obtaining informed consent. First, a spontaneously expectorated sputum was sampled. Then a second sputum sample was collected after induction *via* the nebulization of saline solution (0.9% NaCl) for 15 min. Clinical microbiology data were obtained from the cultivation of sputum samples collected from the same patients during a previous within-week consultation.

### Protein extracts and proteolysis

Sputum samples were processed after sampling (within less than 4 h at room temperature). Samples were filtered through a 70 μm cell strainer (Falcon) and centrifuged at 5,000× *g* for 3 min to reduce the viscosity of the mucus matrix. The samples were then centrifuged at 10,000× *g* for 10 min. After discarding the supernatant, the cell pellets containing the microbial fraction were homogenized in lithium dodecyl sulfate (LDS) 1X lysis buffer (60 μl per mg of pellet) containing 26.5 mM Tris base, 0.5% lithium dodecyl sulfate (w/v), 2.5% glycerol (w/v), 0.13 mM EDTA, 0.06 mM SERVA Blue G-250, 0.04 mM phenol red, buffered pH 8.5 and supplemented with 5% beta-mercaptoethanol (v/v; Mappa et al., 2021). Samples were incubated for 10 min at 99°C in a thermomixer (Eppendorf) and then sonicated 5 min in an ultrasonic water bath (VWR ultrasonic cleaner). The suspensions were transferred into 0.5 ml Screw Cap microtubes (Sarstedt) containing 200 mg of a mixture of beads (0.1 mm silica beads, MP Biomedicals, 0.1 and 0.5 mm glass beads, Bertin Technologies) as recommended for obtaining the lysis of a wide range of microorganisms (Hayoun et al., 2019). Cell disruption was operated at 10,000 rpm for 10 cycles of 30 s, with 30 s of pause between each cycle using a Precellys Evolution instrument (Bertin Technologies). Samples were centrifuged at 10,000× g for 1 min. The resulting supernatants containing the protein extracts were stored at −20°C until further use. The samples were heated for 5 min at 99°C. Then, a volume of 25 μl was loaded on a NuPAGE 4–12% Bis-Tris gel. Denatured proteins were subjected to 5 min SDS-PAGE migration. The proteins were stained for 10 min with Coomassie SimplyBlue SafeStain (Thermo Fisher Scientific) prior to in-gel trypsin proteolysis performed as described (Rubiano-Labrador et al., 2014).

### Tandem mass spectrometry

The peptides were analyzed with a Q Exactive HF tandem mass spectrometer (ThermoFisher Scientific) coupled to an Ultimate 3000 Nano LC System (ThermoFisher Scientific). Peptides (200 ng) were injected onto a reverse-phase Acclaim PepMap 100 C18 column (3 μm, 100 Å, 75 μm id × 500 mm), desalted, and then resolved at a flow rate of 0.2 μl/min with a 120 min gradient of CH_3_CN (4–25% for 100 min, then 25–40% for 20 min) in the presence of 0.1% HCOOH. The tandem mass spectrometer was operated in data-dependent mode. Briefly, a scan cycle was initiated with a full scan of peptide ions in the ultra-high-field Orbitrap analyzer, followed by the selection of a single precursor, its dissociation in high-energy collisional mode, and scan of its fragments. MS/MS successive scans on each of the 20 most abundant precursor ions were conducted (Top20 strategy), selecting parent ions with potential charge states of 2+ or 3+ and a dynamic exclusion of 10s. Eventually, a list of excluded masses was activated. Full scan mass spectra were acquired from 350 to 1,800 *m/z* with an automatic gain control (AGC) target set at 1.74 × 10^6^ ions and a resolution of 60,000. MS/MS scans were acquired at a resolution of 15,000 when the AGC target reached 1 × 10^5^ ions with a threshold intensity of 83,000. Each sample was analyzed once under these MS conditions to generate a mass exclusion list and three additional times under these conditions but using a mass exclusion list (Supplementary Table S2) to guide spectra acquisition.

### Data interpretation

MS/MS spectra were assigned to peptide sequences using Mascot Daemon 2.6.1 (Matrix science) as previously described (Grenga et al., 2022). Briefly, the parameters for assignation were: full trypsin specificity, maximum of one missed cleavage, 2+ and 3+ peptide charges, mass tolerances on the parent ion of 3 ppm, and 0.02 Da on the MS/MS, carboxyamidomethylation of cysteine (+57.0215) as static modification, and oxidation of methionine (+15.9949) as a dynamic modification. The starting database was NCBInrS, an in-house assembled subset of the NCBInr database downloaded the 3rd of January 2018 as ftp://ftp.ncbi.nlm.nih.gov/blast/db/FASTA/nr.gz, with filtering to retain one representative taxon per species belonging to the superkingdoms Bacteria, Archaea and Eukaryota. The NCBInrS database includes 50,080,649 protein sequences from 199,297 taxa, totaling 20,054,069,172 amino acid residues. At each taxonomical rank, the Taxon-Spectrum Matches (TSMs) as first defined in Pible et al. (2020) and the number of taxon-specific peptide sequences were measured for each identified taxon. Families were validated in the first-round search if identified with (i) more than a given family-specific peptides or (ii) more than a given number of additional TSMs explaining the presence of this lineage. The thresholds for these two parameters were established depending on the amount of signals in the dataset and the superkingdom considered. The thresholds are indicated for each dataset (Table 1). Together with all their descendants, all the representatives of these families were extracted from the full NCBInr database (downloaded on 2018-01-08). This new database was queried with the same settings except for the maximum missed cleavages and the mass tolerance on the parent ion set to 2 and 5 ppm, respectively. Genera were validated in the second-round search if identified with (i) more than a given genus-specific peptides or (ii) more than a given number of additional TSMs explaining the presence of this lineage. These signal-dependent thresholds are indicated in Table 1. All descendants of these genera were extracted from NCBInr for a third Mascot search performed with the same settings. At this stage, peptides and proteins were identified with a false discovery rate (FDR) of 1%. The FDR was estimated with a search against a decoy database with the Mascot engine. Proteins were grouped into protein families, and their abundance was reported based on parsimony.

**Table 1:** Metaproteomic data of spontaneous versus induced sputum samples using a mass-exclusion list strategy by nanoLC-MS/MS.

		Patient 1		Patient 2		Patient 3	
		Spontaneous	Induced	Spontaneous	Induced	Spontaneous	Induced
	MS/MS spectra	48,300	59,995	35,794	65,091	54,740	53,871
Step 1	PSMs^a^	13,955	18,760	10,382	23,901	16,635	16,051
	Family #SpePep threshold	2	2	2	2	2	2
	Family #TSM threshold	4	5	4	5	5	5
	Tax ids DB step 2	5,362	5,048	5,236	7,848	6,208	5,635
Step 2	PSMs^a^	12,659	17,925	8,954	23,312	15,115	14,612
	Genera #SpePep threshold	1	1	1	1	1	1
	Genera #TSM threshold	5	5	5	5	5	5
	Tax ids DB step 3	2,920	2,787	2,448	4,853	2,731	2,469
Step 3	PSMs^a^	10,376	15,272	7,193	19,978	13,018	12,542
	Attribution rate (%)	21.9	25.6	16.5	31.5	26.4	24.1
	#SpePep^b^ Eukaryota	3,574	5,359	2,264	7,087	2,435	3,264
	#SpePep Bacteria	119	234	106	313	116	135
	Eukaryotic signal (% Norm. specific peptides)	96.8	95.8	95.5	95.8	95.5	96.4
	Bacterial signal (% Norm. specific peptides)	3.2	4.2	4.5	4.2	4.5	3.6
	Number of identified proteins	743	1,102	758	1,653	579	679
	Number of peptides	3,812	5,666	3,397	7,693	2,733	3,538
	Number of proteins attributed to *Homo sapiens*	574	834	577	1,240	385	483
	Number of peptides attributed to *Homo sapiens*	3,513	5,211	3,084	7,032	2,478	3,263
	Number of microbial proteins	122	200	130	298	154	158
	Number of microbial peptides	191	310	191	421	197	209
	Number of proteins attributed to other Eukaryota	47	68	51	115	40	38
	Number of peptides attributed to other Eukaryota	108	145	122	240	58	66

The values reported are the averaged values from three technical replicates.

a *PSMs, peptide-spectrum matches.*

b *#SpePEP, specific peptides.*

Taxonomical differential analysis of the microbial composition of spontaneous and induced sputum was assessed in terms of taxa abundance by using the R package *metacoder* (Foster et al., 2017). Reactome Gene Sets (RHSA)-enrichment was done *via* Metascape (Zhou et al., 2019). Statistically enriched (FDR ≤ 0.01) RHSA terms were identified by searching a database containing the respiratory tract-specific proteome from the Human Protein Atlas Project version 20.0 (downloaded on the 2020-11-16 from https://www.proteinatlas.org/). The sum of the normalized spectral abundance factor (NSAF) of each human protein contributing to the functional Reactome terms was used as their relative abundance. The NSAF values have been calculated as described previously (Christie-Oleza et al., 2012). Within each phylum, the sum of spectral counts of the proteins for a taxon were used to assign abundance values to protein taxon. The microbial proteins were KEGG-annotated using the online tool GhostKOALA^1^ (Kanehisa et al., 2016).

## Results

### Optimization of tandem mass spectrometry for the proteotyping of *CF* sputa

**Figure 1:**
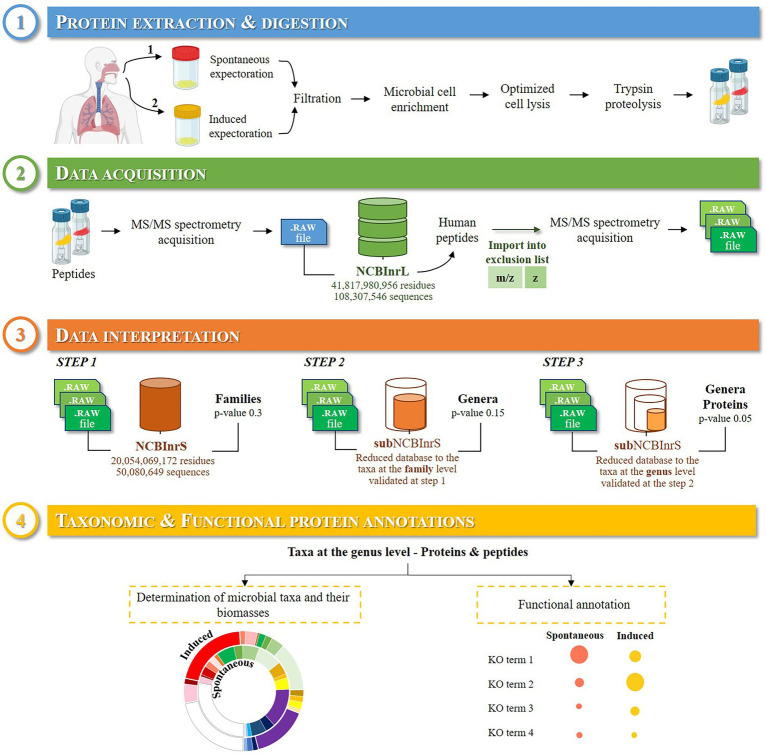
Experimental workflow of the study. ① Paired spontaneous and induced sputum samples collected from *CF* patients were filtered to reduce their viscosity and submitted to differential centrifugation to concentrate the microorganisms. The proteins extracted were submitted to a short in-gel migration and digested into peptides using trypsin. ② The resulting peptides were analyzed by nanoLC-MS/MS. ③ Results were interpreted in a three-step cascaded search to retrieve the taxonomic and functional protein annotations ④.

To assess the impact of the sample type on the investigation of the respiratory microbiota, pairs of spontaneous (SS) and induced sputum (IS) samples collected from three *CF* adult patients during a follow-up consultation (Table 2) were analyzed following the approach described in Figure 1. To retrieve the taxonomic and functional protein annotations, the acquired mass spectrometry data were interpreted in a three-step cascaded search. Specifically, the first two rounds of the search were used to optimize the identification of taxa in the sample. While the first round was used to refine the search to the Life’s branches more likely represented, the second round identified the genera that best explained them and confirmed whether they were present in the sample. The third round performed a more classical protein search on the most adequate database comprising only the identified genera. An average of 30.5 ± 4.5% of the MS/MS spectra were assigned to peptide sequences (Table 3). A total of 16,550 ± 4,627 TSMs were obtained and used to confidently identify the taxa detectable in the samples (value of *p* ≤ 0.05).

**Table 2:** Patient characteristics and results of spontaneous sputum sample cultures.

	Patient 1	Patient 2	Patient 3
Sex	F	F	M
Age (yr)	23	39	35
Antibiotic therapy	Linezolid Aztreonam Colistin	Linezolid Tobramycin Colistin	Ciprofloxacin Trimethoprim-sulfamethoxazole Colistin
Pathogens^a^	*Escherichia coli* (2×10^6^ CFU/ml) *Staphylococcus aureus* (4.10^5^ CFU/ml) Candida albicans^*^	*Staphylococcus aureus* (2.10^4^ CFU/ml) *Pseudomonas aeruginosa* (3.10^6^ CFU/ml) Aspergillus flavus^*^ Candida dubliniensis^*^	*Morganella morganii* (2×10^4^ CFU/ml) *Proteus mirabilis* (8×10^2^ CFU/ml) *Staphylococcus aureus* (7×10^5^ CFU/ml) Mycobacterium avium^*^ Trichosporon mycotoxinivorans^*^ Aspergillus fumigatus^*^ Candida albicans^*^

a Sputum pathogens have been cultured, identified and quantified according to national guidelines for the microbiological analysis of sputum samples of CF patients (Société Française de Microbiologie, 2018).

* Species load were not determined during analysis.

**Table 3:** MS/MS interpretation for spontaneous and induced sputum samples.

	Patient 1		Patient 2		Patient 3	
	Spontaneous	Induced	Spontaneous	Induced	Spontaneous	Induced
MS/MS spectra	47,406	62,164	47,580	67,355	51,122	54,658
PSMs^a^	9,748	17,825	15,681	24,205	16,982	17,268
Attribution rate (%)	20.6	28.7	33.0	35.9	33.2	31.6
#SpePep Eukaryota	2,860	5,765	3,036	8,449	3,158	3,955
#SpePep^b^ Bacteria	82	224	147	297	112	129
Eukaryotic signal (% Norm. specific peptides)	97.2	96.2	95.4	96.6	96.6	96.8
Bacterial signal (% Norm. specific peptides)	2.8	3.8	4.6	3.4	3.4	3.2
Total number of proteins	974	1,581	905	2,585	870	1,063
Total number of peptides	2,436	4,641	2,472	6,911	2,701	3,411
Number of proteins attributed to *Homo sapiens*	498	918	405	808	567	624
Number of peptides attributed to *Homo sapiens*	2,180	4,118	2077	4,981	2,599	3,199

a *PSMs, peptide-spectrum matches.*

b *#SpePEP, specific peptides.*

**Figure 2:**
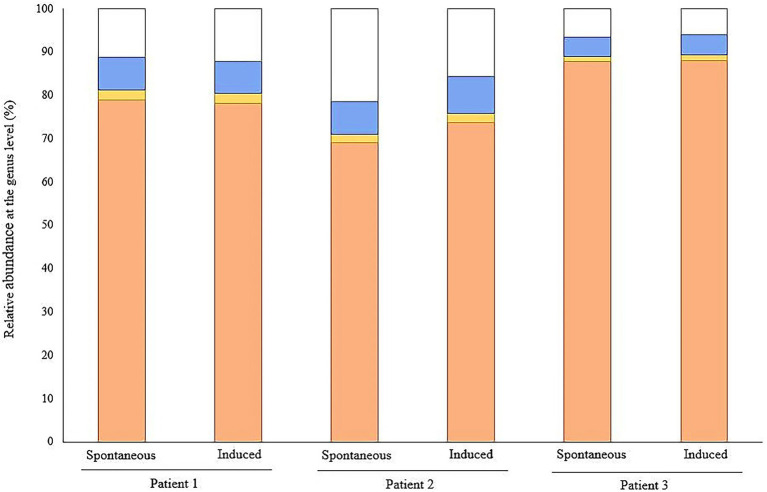
Relative protein abundance according to their human, fungal or bacterial origin. The stacked bar graphs show the relative abundance (%) that corresponds to the normalized TSM signals at the genus level from the triplicates of each sample for human signal (orange), fungal taxa (yellow), bacterial taxa (blue) and the non-attributed signals at the taxonomic level of genus (white).

**Figure 3:**
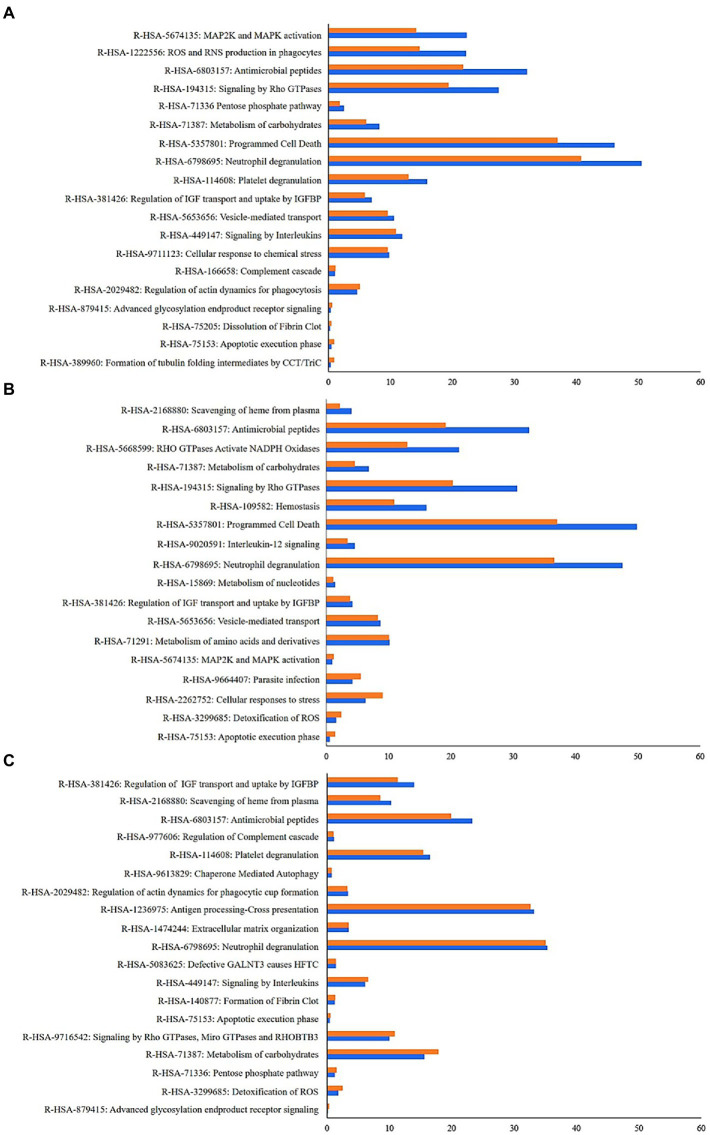
Enrichment functional analysis of the human proteins identified in both induced sputum (IS) and spontaneou sputum (SS) samples from the three patients. The Representative Reactome Gene Sets terms identified by annotating human proteins from SS and IS samples are indicated in blue and orange, respectively. Their relative abundance corresponds to the sum of the normalized spectral abundance factor of each human proteins contributing to the functional reactome terms. **(A)** Patient 1, **(B)** Patient 2 and **(C)** Patient 3.

Overall, a large proportion of the recorded signal was attributed to the superkingdom Eukaryota with more than 95% of the spectra assigned to human proteins (Table 3; Figure 2). A total of 1,982 human proteins were identified, 198 of which were common to the SS samples while 325 proteins were shared in the IS ones (Supplementary Table S1). The most abundant among the latter were the alpha-amylase and trypsinogen precursors, S100-A9 protein, serum albumin, and the polymeric immunoglobulin receptor. Their proteotypic peptides accounted for 43% of the total Peptide-Spectrum Matches (PSMs). The functional annotation of the identified human proteins revealed that key processes and pathways linked to the *CF*-affected airways were commonly identified in both IS and SS samples (Figure 3).

To improve the detection of microbial peptides, an exclusion list containing 950 *m/z* values corresponding to the 450 most abundant out of 859 peptides of human origin commonly found in all the samples was used to guide spectra acquisition in a second nanoLC-MS/MS analysis of each peptide pool in analytical triplicates (Supplementary Table S2). Results from the analysis of the three technical replicates per sample are described in Table 1. While the overall number of MS/MS spectra and PSMs before and after implementing the mass-exclusion list were comparable, the use of the mass-exclusion list enhanced the detection of the microbial signal present in the samples with on average 41.8 ± 4.1% PSMs belonging to microbial genera.

### Spontaneous and induced sputum samples hold similar information regarding the microbial community structure

**Figure 4:**
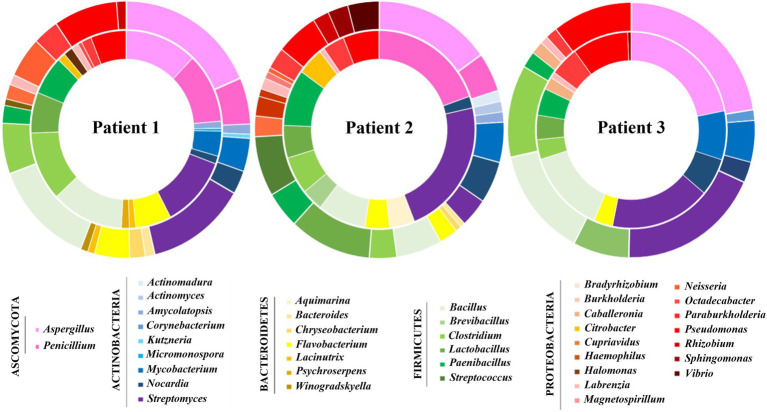
Relative abundance of microbial taxa at the genus level in spontaneous sputum (SS) versus induced sputum (IS) samples determined following the mass-exclusion list strategy. The relative abundance (%) corresponds to the normalized TSM signals at the genus level. The inner circle indicates the relative abundance of the microbial taxa found in SS sample whereas the outer circle corresponds to the results for IS sample. Each value corresponds to the mean of the analytical triplicates per sample.

The relative abundance of each microbial genera was estimated by normalizing their assigned TSMs by the sum of TSMs attributed to all microbial genera. A total of 38 different microbial genera were confidently identified in the 6 samples. Among them, 9 bacterial genera were found systematically in the three pairs of samples: *Streptomyces*, *Bacillus*, *Clostridium*, *Pseudomonas, Nocardia*, *Flavobacterium*, *Paenibacillus*, *Labrenzia*, and *Paraburkholderia* (Table 4; Figure 4). The four first cited genera were the most abundant and collectively represented on average 40.4 ± 7.3% of the bacterial biomass. To evaluate the impact of the sampling method on the abundance of the microbial components in sputum samples, a comparison analysis was performed between the microbial genera found in pairs of SS and IS samples. No significant difference was observed between the abundances of these microbial genera in SS and IS from Patients 1 and 3. Regarding the two samples from Patient 2, *Penicillium* was the only genus significantly found more abundant (value of *p* ≤ 0.05) in the SS compared to the IS. Taxa that were differentially identified according to the sample were *Micromonospora* identified in the spontaneous condition only (SS1), *Neisseria* and *Cupriavidus* observed in induced sputa (IS1 and IS3, respectively), *Burkholderia* uniquely found in SS1 and SS2, and *Corynebacterium* found only in IS3. Interestingly, the methodology is able to detect not only bacteria but also fungi. Among the fungi, both *Aspergillus* and *Penicillium* were identified in sputum samples from Patient 1. These two fungal genera were found in the IS of Patient 2, but only *Penicillium* was observed in the SS2. *Aspergillus* was the only fungal genus found in samples from Patient 3.

**Table 4:** Microbial genera systematically identified in the three patients.

	Patient 1		Patient 2		Patient 3	
	Spontaneous	Induced	Spontaneous	Induced	Spontaneous	Induced
*Nocardia*	1.3 ± 0.2	2.9 ± 0.2	1.9 ± 0.4	5.3 ± 1.2	6.2 ± 0.8	2.6 ± 0.5
*Streptomyces*	11.7 ± 1.7	13.1 ± 3.3	22.3 ± 8.3	3.6 ± 0.4	17.1 ± 2.8	18.7 ± 3.1
*Flavobacterium*	6.0 ± 1.4	4.6 ± 1.2	4.1 ± 0.3	2.4 ± 0.4	3.2 ± 0.3	7.3 ± 1;6
*Bacillus*	12.3 ± 2.5	13.4 ± 4.2	8.1 ± 3.2	5.9 ± 1.2	13.8 ± 0.6	13.9 ± 2.6
*Clostridium*	11.3 ± 2.3	6.4 ± 1.2	6.1 ± 1.5	3.5 ± 0.6	3.2 ± 0.9	11.7 ± 1.3
*Paenibacillus*	6.4 ± 1.4	2.2 ± 0.9	9.2 ± 2.3	4.6 ± 0.8	4.5 ± 1.1	2.1 ± 0.4
*Labrenzia*	1.2 ± 0.2	1.3 ± 0.6	0.8 ± 0.1	1.4 ± 0.4	1.0 ± 0.6	1.0 ± 0.8
*Paraburkholderia*	1.7 ± 0.3	3.4 ± 0.3	3.7 ± 0.4	2.7 ± 0.9	4.8 ± 0.7	1.5 ± 0.6
*Pseudomonas*	6.0 ± 2.4	8.4 ± 2.4	6.1 ± 1.2	5.5 ± 3.2	9.5 ± 1.8	10.5 ± 2.6

The relative abundance (%) corresponds to the normalized TSM signal at the genus level. The values given correspond to the mean of the technical triplicates analyzed for each sample.

*CF* clinical management is routinely performed by the cultivation of sputum samples on a panel of agar media, including selective and enriched media, followed by identification of targeted pathogens *via* morphological analysis, Gram stain, MALDI-TOF or, less frequently, PCR on the positive cultures. Here, because of the volumes required and the limited amount of sputum collected, the clinical microbiology data were obtained from spontaneous sputum samples collected 8 days before those profiled by tandem mass spectrometry. Culturing the sputum samples of the 3 *CF* patients allowed identifying a total of 14 strains belonging to 9 microbial genera (Table 2). Among them, the metaproteomics approach allowed the detection of known *CF* pathogens such as *Pseudomonas*, *Mycobacterium,* and *Aspergillus* but not *Staphylococcus* and *Candida*. Concomitantly, 28 and 12 additional microbial genera were identified in the IS and SS samples, respectively, following sample profiling by tandem mass spectrometry (Figure 4).

### Microbial signal can be profiled to provide a functional view of the microbiota activity

To describe the functional composition of the microbiota, microbial proteins identified in each sample were annotated and assigned to 343 KO (KEGG Orthology) terms. These were grouped into 49 KO parent-terms. The most important categories of annotated microbial proteins, based on the abundance and number of KO-terms, are involved in pathways relevant to airway infection such as evasion from the host immune system or microbial pathogenicity. KO-terms responsible for multidrug resistance (K18888, K04771) were identified in SS from Patient 1 (SS1) and quorum sensing (K01657) in IS from Patient 1 (IS1). KO-terms (K02034, K02035) involved in biofilm formation and peptide/nickel transport were found in SS1 and SS from Patient 2 (SS2). KO-terms involved in lipopolysaccharide biosynthesis and transport (K04077), lipoarabinomannan biosynthesis (K04043) and chemotaxis (K03412) were both found in the sputa, whether SS or IS, from patients 2 and 3. Additionally, KO-terms for Type IV secretion system (K20527) and metallic and iron transport system (K02012) were, respectively, found in SS and IS from Patient 3 (SS3 and IS3).

**Figure 5:**
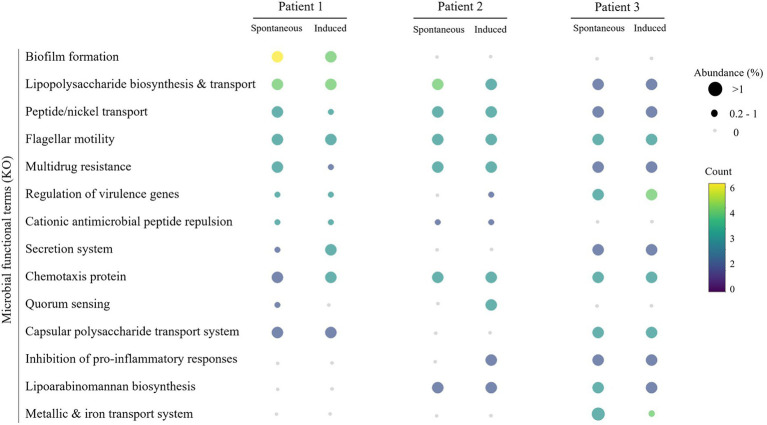
Functional annotation of the microbial proteins found in spontaneous and induced sputum samples. The relative abundance of each category corresponds to the sum of the relative abundance of each KEGG Orthology (KO) term of interest of the related category, which is indicated by the size of the dot. The color scale corresponds to the number of KO terms of interest involved in each category.

The annotated microbial proteins of interest highlighted the role of specific microbiota components in the colonization of the *CF* respiratory tract (Figure 5; Table 5). Among them, we detected peptide/nickel transport system permease protein and its substrate, preprotein translocase subunit SecA and a large repetitive protein required for biofilm formation and host colonization *via* virulence and antibiotic resistance in SS1 and IS1. In addition, proteins involved in cationic antimicrobial peptide repulsion (SecA, cheY, degP) and multidrug resistance (ABCG2.PDR, efrB) were detected in both SS and IS samples from Patients 1 and 2. Biofilm formation and host colonization are increased with the production of chemotaxis and flagellar motility proteins found in all the samples. The molecular chaperones DnaK (SS2 and IS2) and GroEL (SS2 and IS2, SS3 and IS3) are responsible for protein protection or degradation due to the oxidative stress mainly established by the host immune system. Additionally, metallic and iron transport systems, which are greatly involved in bacterial virulence, were detected (AfuA, FhuB and EcfA2) both in SS3 and IS3.

**Table 5:** Functional annotation of the microbial proteins of interest.

KO terms	Name	Description	Categories	Spontaneous sample	Induced sample
K02034	ABC.PE.P1	Peptide/nickel transport system permease protein	Biofilm formation Peptide/nickel transport	SS1 SS2	IS1 IS2
K02035	ABC.PE.S	Peptide/nickel transport system substrate-binding protein	Biofilm formation Peptide/nickel transport	SS1 SS2 SS3	IS1 IS2 IS3
K03070	secA	Preprotein translocase subunit secA	Biofilm formation Regulation of virulence genes Cationic antimicrobial peptide repulsion	SS1	/
K20276	bapA	Large repetitive protein	Biofilm formation	SS1	/
K09691	ABC-2.LPSE.A	Lipopolysaccharide transport system ATP-binding protein	Lipopolysaccharide biosynthesis and transport	/	IS1
K11444	wspR	Two-component system chemotaxis family, response regulator WspR	Lipopolysaccharide biosynthesis and transport Capsular polysaccharide transport system	SS1	IS1
K04077	groEL	Chaperonin GroEL	Lipopolysaccharide biosynthesis and transport	SS2 SS3	IS2 IS3
K07091	lptF	Lipopolysaccharide export system permease protein	Lipopolysaccharide biosynthesis and transport	SS2	/
K09808	lolC_E	Lipoprotein-releasing system permease protein	Lipopolysaccharide biosynthesis and transport	SS2	IS3
K02031	ddpD	Peptide/nickel transport system ATP-binding protein	Peptide/nickel transport	SS2	IS2
K03776	aer	Aerotaxis receptor	Flagellar motility	/	IS2
K03406	mcp	Methyl-accepting chemotaxis protein	Flagellar motility Chemotaxis protein	SS1 SS2 SS3	IS1 IS2 IS3
K03412	cheB	Two-component system, chemotaxis family	Flagellar motility Chemotaxis protein	SS2 SS3	IS2 IS3
K03413	cheY	Two-component system, chemotaxis family	Flagellar motility Chemotaxis protein	SS1 SS2	/
K08711	ABCG2.PDR	ATP-binding cassette, subfamily G	Multidrug resistance	/	IS1
K18888	efrB	ATP-binding cassette, subfamily B, multidrug efflux pump	Multidrug resistance	SS1	/
K04771	degP	Serine protease Do	Multidrug resistance Cationic antimicrobial peptide repulsion	SS1	IS1
K05658	ABCB1	ATP-binding cassette, subfamily B (MDR/TAP), member 1	Multidrug resistance	SS2	/
K18889	mdA	ATP-binding cassette, subfamily B, multidrug efflux pump	Multidrug resistance	/	IS2
K07679	evgS	Two-component system, NarL family, sensor histidine kinase EvgS	Multidrug resistance	SS3	IS3
K01104	E3.1.3.48	Protein-tyrosine phosphatase	Regulation of virulence genes	SS1	IS1
K11895	impH	Type IV secretion system protein ImpH	Secretion system	/	IS2
K20527	trbB	Type IV secretion system protein TrbB	Secretion system	SS3	IS3
K01657	trpE	Anthranilate synthase component	Quorum sensing	SS1	IS1
K01995	livG	Branched-chain amino acid transport system ATP-binding protein	Quorum sensing	/	IS2
K01996	livF	Branched-chain amino acid transport system ATP-binding protein	Quorum sensing	/	IS2
K09689	kpsT	Capsular polysaccharide transport system ATP-binding protein	Capsular polysaccharide transport system	SS3	IS3
K10107	kpsE	Capsular polysaccharide transport system permease protein	Capsular polysaccharide transport system	SS3	IS3
K04043	dnaK	Molecular chaperone DnaK	Lipoarabinomannan biosynthesis	SS2 SS3	IS2 IS3
K02040	pstS	Phosphate transport system substrate-binding protein	Lipoarabinomannan biosynthesis	SS3	/
K02012	afuA	Iron(III) transport system substrate-binding protein	Metallic and iron transport system	SS3	IS3
K23228	fhuB	Ferric hydroxamate transport system permease protein	Metallic and iron transport system	/	IS3
K16787	ecfA2	Energy-coupling factor transport system ATP-binding protein	Metallic and iron transport system	SS3	IS3

To evaluate the effect of the sampling method on the ability to recover functional information, a comparative analysis of the abundance of the microbial protein functions in SS and IS samples was performed. The relative abundance of KO terms was compared in paired SS and IS samples. No significant difference (value of *p* ≤ 0.05) in the abundance of KO terms was observed between SS and IS in the three patients (Figure 5).

## Discussion

A comprehensive understanding of the taxonomy and functionality of the *CF* airway polymicrobial microbiota could improve infection diagnosis, management, and prediction. We applied an innovative tandem mass-spectrometry-based proteotyping approach to analyze paired spontaneous and induced sputum samples collected from three *CF* patients, revealing the structure of the microbial communities but also uncovering the microbial functions encoded by the most abundant proteins. These data illustrate how a quick analysis of sputum samples can be performed to identify and quantify the main taxa and obtain host-related information. Induced or spontaneous sputum samples were shown to be equivalent for this methodology, paving the road for its wide application.

Although a protocol for metaproteomic analysis of *CF* sputum samples has been previously proposed (Graf et al., 2021), the need to create dedicated databases obtained after 16S RNA gene amplicon sequencing on the sputum sample and the multiple steps for enrichment in microorganisms make the approach incompatible with routine microbiological diagnosis. The protocol described in the present study does not require time-consuming steps of mechanical and enzymatic homogenization, differential centrifugations, and filtrations. Instead, the protocol is easy to perform and delivers a higher percentage of assigned spectra. Furthermore, compared to DNA extraction and sequencing, either 16S RNA gene amplicons or metagenomics, the tandem mass spectrometry-based proteotyping proposed herein does not introduce amplification bias and delivers the whole panorama of microorganisms present in the sample. Last, our approach performs more quickly than time-consuming culture-dependent methods that are stochastics per nature and is more comprehensive.

Characterizing low biomass clinical microbiome is challenging because of the high abundance of the host signal and the lack of comprehensiveness of the database used for the interpretation. This last item is not anymore prominent as almost all pathogens and clinically relevant opportunistic microorganisms have publicly available whole-genome sequences. The remaining difficulty, i.e., the dynamic range of microbial and host proteins in sputum samples, has been tackled by removing the most abundant host proteins in bronchoalveolar lavage fluid (BALF) samples *via* two sequential analytical and bioinformatical workflows based on a custom database comprising human and microbial protein sequences expected in BALF (Jagtap et al., 2018). Here, we have developed another option based on a list of excluded mass/charge signals to avoid the tandem mass spectrometer spending too much time on these uninformative signals. While most of these host signals arise from conserved human proteins, whether the list established here could be used for other studies or should be revisited to be more specific of the new samples should be carefully evaluated. We also increased the ratio of interpreted MS/MS spectra by applying a smart strategy to reduce the search space and adjust the database for each sample to the microorganisms present in the sample. This approach is well adapted for interpreting metaproteomics data without *a priori* (Bassignani et al., 2021; Jouffret et al., 2021; Grenga et al., 2022). Here, we obtained the list of taxa present in the samples at the genus level in most cases, but the precision of the identification can be theoretically increased at the species level as soon as more signals are recorded on the same samples. Because of the higher abundance of the host proteins compared to the microbial ones and of the mass spectrometry sampling effect, as expected, the peptides/protein ratio is higher for *H. sapiens* (6) compared to microorganisms (1.4) and other Eukaryota (2). The best current practice in metaproteomics recommends informing the protein sequences database with data obtained by metagenomics or 16S rRNA amplicon sequencing performed on the same samples (Van Den Bossche et al., 2021b). However, this would require (i) more biological material, while the sputum samples could be difficult to obtain, and (ii) more time, while quick analysis of clinical samples is of utmost interest for the medical diagnostic. Our approach involves a sample-specific database construction taxonomically-informed, with the difference that the taxonomical information is from the dataset itself instead of relying on additional metataxonomics or metagenomics data with their own shortcomings. The most striking characteristic of the methodology is the possibility to estimate the relative abundance of the taxa identified, gaining further insight into the microbial community structure and allowing monitoring its dynamics longitudinally through the comparison of sample profiles. For this, the low microbial signal quantitation is based on the TSM concept, first described by Pible et al. (2020). Because of the development of new quantitation strategies, such as the ones based on data-independent acquisition (Aakko et al., 2020), the accuracy of this quantitative methodology could be even further improved.

When interpreting the recorded signals, host proteins are identified. Several assigned functions to this host signal were previously and directly linked to the *CF*-affected airways, such as complement cascade, signaling by interleukins and defective CFTR, apoptotic execution phase and programmed cell death (Soleti et al., 2013), scavenging of heme (Tyrrell and Callaghan, 2016), autophagy (Bodas and Vij, 2019; Zhao et al., 2020), cellular response to stress or antimicrobial peptides (Laube et al., 2006; Lecaille et al., 2016). Among these functionalities, apoptosis and programmed cell death greatly participate to the onset of the disease *via* multiple mechanisms that could be defective for the neutrophils or increased in epithelial cells, and then constantly contribute to the inflammatory state. In this context of inflammation and infection, scavenging of heme is important in heme management for heme-iron recycling but also in response to oxidative stress and in vesicle-mediated transport for the reticulum endoplasmic trafficking that are greatly imbalanced. With these conditions and stresses due to CFTR default that facilitates the colonization of the lung by microbial pathogens and the subsequent potential establishment of chronic infections leading to tissue damage, the airways provide a myriad of mechanisms of defense, notably lung antimicrobial peptides present at the surface of the epithelial cells which are markedly increased to prevent or in response to microbial colonization and infections. The insights of these functionalities highlighted by the proposed approach could be more deeply analyzed.

Clinical *CF* management is currently based on classical culture-based approaches to identify whether the most common *CF* pathogens are present in the sputum sample, and quantify them. However, this *a priori* approach suffers several limitations despite being a reference method allowing strain recovery and further antimicrobial susceptibility testing. Here, we compared the identified pathogens detected *via* morphological analysis after a cultivation step of sputum samples in the framework of the microbiological monitoring of *CF* patients and the tandem mass spectrometry proteotyping. Some of the cultured microorganisms, such as *Escherichia coli* in Patient 1, *Staphylococcus aureus* and *Candida albicans* in the three patients, were not detected by the described metaproteomics approach, probably due to the 8 days delay and the antibiotic therapies administered between classic microbiological assessment and sputum sample collection for the metaproteomics analysis. Above all, our metaproteomics-based approach without *a priori* allows enlarging the vision of the microbiota representatives including potential pathogenic microorganisms for *CF* patients and opportunistic ones. With the emergence of new *CF* pathogens and the identification of unusual species (Marchandin, 2012; Menetrey et al., 2021), depending on the specific environment and exposition of patients, the approach presented here appears highly complementary to the more classic microbiological approaches and could help clinical management of airway infections with its additional valuable information. The clinical relevance of the taxa detected by our mass spectrometry-based approach remains to be clarified by more extensive and longitudinal studies of the *CF* lung microbiota. On the other hand, the lack of detection of some taxa identified by culture in the previous week at various loads also has to be clarified to optimize the approach, as corresponding peptides/proteins might have been excluded from analysis due to too stringent analysis criteria (step 3 in Figure 1) after mass-exclusion list implementation.

Last but not least, the lung microbiota is a complex microbial ecosystem in which each microorganism may play a key role or become a major player in the course of the infection. Identifying specific functionalities, such as virulence factors in specific microorganisms may lead to a better understanding of the lung microbiota’s relation to disease evolution and outcome and could be used as prognostic markers of the onset of infection or exacerbations in *CF* patients. Here, we have specifically pointed out proteins known to be involved in microbial pathogenicity and virulence. Proteins for iron sequestration, flagellar motility and antibiotic resistance, and biofilm growth are all virulence factors that play a major role during bacterial infection to counteract the host defense and clearance mechanisms.

With the perspective of the application of the methodology for diagnostic in the clinic, we evaluated to 5 h the time to perform all the experimental operations and interpretation: sample preparation (2 h), mass spectrometry (1 h), and automatized interpretation (2 h). Such operations could be further optimized with specific efforts of industrial development to obtain the results more quickly and automatize the operations. Due to the known high variability between *CF* patients and the unicity of each patient’s microbiota (e.g., specific mutations in the CFTR gene and the individual patient treatment regimens), the analysis of a larger sample size may have revealed additional microorganisms/functions. Still, it would not have impacted the comparative analysis of each paired sputum sample. Conversely, a much larger sample number will be required to investigate and validate the biological significance of the metaproteomics results.

In summary, the sputum microbiota of three *CF* patients presents a personal signature with no significant difference in microbial features and activity in terms of abundance between paired spontaneous and induced sputum samples allowing a comprehensive characterization of the *CF* microbiota, including bacteria and fungi components, whatever the sampling method. By applying our innovative metaproteomics-based approach, we can detect the abundant microbial genera, including specific *CF* pathogens and the less explored and emerging ones. This additional information is a valuable complement to the classical microbiological diagnosis of *CF* airway infection and can be provided quickly. This is a major achievement as an unbiased view of the microbiome could be highly complementary and relevant for the clinical management of *CF* patients and for further personalized and predictive medicine by improving knowledge about the host-pathogen dynamics and *CF* pathophysiology.

## Data availability statement

The mass spectrometry proteomics data have been deposited to the ProteomeXchange Consortium via the PRIDE (Perez-Riverol et al., 2022) partner repository with the dataset identifier PXD034628 and 10.6019/PXD034628.

## Ethics statement

The studies involving human participants were reviewed and approved by Centre de Ressources et de Compétences de la Mucoviscidose in the University Hospital of Montpellier (France). The patients/participants provided their written informed consent to participate in this study.

## Author contributions

JA, RC, and LG designed the study. RC collected the samples and clinical data. PH performed sample preparation for metaproteomic analysis. PH, OP, HM, KC, and LG conceived and performed data and statistical analyses. PH, JA, and LG drafted the manuscript. HM, JA, RC, and LG critically revised the manuscript. All authors contributed to the article and approved the submitted version.

## Funding

This work was funded in part by the ANR program “Phylopeptidomics” (grant number ANR-17-CE18-0023-01).

## Conflict of interest

The authors declare that the research was conducted in the absence of any commercial or financial relationships that could be construed as a potential conflict of interest.

## Publisher’s note

All claims expressed in this article are solely those of the authors and do not necessarily represent those of their affiliated organizations, or those of the publisher, the editors and the reviewers. Any product that may be evaluated in this article, or claim that may be made by its manufacturer, is not guaranteed or endorsed by the publisher.

## Footnotes

^1^ https://www.kegg.jp/ghostkoala/
